# The association of the combined triglyceride-glucose and frailty index with chronic liver disease: evidence from the CHARLS study

**DOI:** 10.1186/s12876-026-04818-1

**Published:** 2026-04-15

**Authors:** Xiaohan Huang, Di Wu, Zeyu Tang, Luzhou Xu, Zhaowei Shan

**Affiliations:** 1 The First School of Clinical Medicine, Nanjing University of Chinese Medicine, Nanjing, Jiangsu China; 2 Department of Gastroenterology, Affiliated Hospital of Nanjing University of Chinese Medicine (Jiangsu Province Hospital of Chinese Medicine), Nanjing, Jiangsu China

**Keywords:** Triglyceride-glucose index, Frailty index, Chronic liver disease, Prospective cohort study, Middle-aged and older adults

## Abstract

**Background:**

The triglyceride-glucose (TyG) index is a recognized marker of insulin resistance, whereas the frailty index (FI) reflects cumulative physiological decline. However, the combined effect of metabolic dysfunction and frailty—referred to as the TyG-Frailty Index (TyGFI)—has not been systematically evaluated. This study aimed to examine the association between TyGFI, analyzed as both continuous and categorical variable, and the risk of chronic liver disease (CLD).

**Methods:**

Baseline and follow-up data were collected from the 2011 and 2015 waves of the China Health and Retirement Longitudinal Study (CHARLS). Multivariable logistic regression models were utilized to evaluate the association between the TyGFI and the risk of CLD, adjusting for demographic characteristics, lifestyle factors, and clinical variables. Restricted cubic spline (RCS) and subgroup analyses were conducted to assess nonlinear dose - response relationships and interaction effects. Sensitivity analyses were performed to evaluate the robustness of the findings.

**Results:**

A total of 7,417 participants were included, of whom 265 developed CLD during follow-up. Elevated TyGFI levels were significantly associated with an increased risk of CLD after multivariable adjustment. Participants in the highest TyGFI quartile exhibited the greatest risk of CLD (OR = 3.19, 95% CI: 2.16–4.80, *p* < 0.001). Each unit increase in TyGFI was associated with a 38% higher odds of incident CLD (OR = 1.38, 95% CI: 1.23–1.45, *p* < 0.001). RCS analysis demonstrated a consistent nonlinear dose-response relationship. ROC analysis showed that TyGFI had moderate discriminative ability for CLD (AUC = 0.64). No significant interaction was observed across subgroups, and sensitivity analyses further confirmed the robustness of the findings.

**Conclusion:**

TyGFI is independently associated with an increased risk of incident CLD and demonstrates modest discriminative ability. By integrating metabolic and functional dimensions, TyGFI may serve as a complementary indicator for risk stratification in middle-aged and older adults, potentially supporting the early identification of individuals at increased risk of CLD.

**Supplementary Information:**

The online version contains supplementary material available at 10.1186/s12876-026-04818-1.

## Introduction

Chronic liver disease (CLD) refers to hepatic disorders characterized by inflammation, necrosis, and fibrosis of the liver due to various etiologies, persisting for more than six months [1]. CLD encompasses a spectrum of conditions, including viral hepatitis, autoimmune liver disease, alcoholic hepatitis, and metabolic dysfunction-associated steatotic liver disease (MASLD) [2]. According to statistics, over 1.6 billion people worldwide are affected by CLD, making it one of the major global disease burdens [3, 4]. MASLD, as a major type of CLD, is associated with an increased prevalence of obesity, diabetes, and metabolic syndrome [5, 6]. With the accelerating pace of population aging, the incidence and mortality of CLD are expected to further increase, highlighting the need for effective tools for early risk assessment and intervention.

The triglyceride-glucose (TyG) index, calculated from fasting triglyceride and glucose levels, has been validated as a specific surrogate indicator of insulin resistance [7]. Increasing evidence suggests that elevated TyG index are associated with the development of various chronic diseases [8–10]. Recent studies have highlighted the predictive value of the TyG index in hepatology, revealing its correlations with MASLD/NAFLD, liver fibrosis, and various other liver conditions [11–14]. Meanwhile, the frailty index (FI) is a validated assessment tool for determining physiological decline. It evaluates overall health by capturing the build-up of health deficits across several areas, such as physical function, comorbidities, cognitive ability, and mental health [15]. FI reflects reduced physiological reserve and increased vulnerability to stressors and has been associated with adverse outcomes such as falls, disability, and increased morbidity [16]. Recent evidence suggests that frailty is associated with an increased risk of CLD, including MASLD, cirrhosis, and liver-related mortality, and predicts poor prognosis in advanced liver disease [17–19]. The FI is further recognized as an independent predictor of adverse outcomes in cirrhotic patients [20].

However, existing research has primarily concentrated on the independent effects of the TyG index or FI on CLD. For instance, several studies have demonstrated the predictive value of the TyG index for MASLD or liver fibrosis without incorporating age-related frailty indicators [11–13], whereas studies focusing on frailty and liver-related outcomes seldom consider metabolic dysfunction [17, 20]. This gap may limit the accuracy of risk stratification in aging populations. Metabolic stress (reflected by TyG) and physiological vulnerability (reflected by FI) often coexist in middle-aged and older adults and may jointly contribute to CLD development through mechanisms such as chronic inflammation and oxidative stress. In addition, traditional liver disease prediction models, such as the fibrosis-4 (FIB-4) index and the NAFLD Fibrosis Score (NFS), primarily rely on liver enzymes and fibrosis-related parameters, while rarely incorporating functional or aging-related factors. However, in middle-aged and older adults, declining physiological reserves and a high burden of comorbidities can significantly influence disease susceptibility and prognosis. Consequently, their applicability for risk stratification in aging populations may be limited [21, 22]. Although recent studies have investigated composite indices combining the TyG index and FI in cardiovascular outcomes, their relevance to CLD remains largely unexplored [23, 24].

Therefore, our study aims to construct and evaluate a TyG–Frailty Index (TyGFI) by integrating the TyG index and FI and to explore its association with incident CLD. Using data from the China Health and Retirement Longitudinal Study (CHARLS), a nationally prospective cohort, we focused on middle-aged and older adults (≥ 45 years). This study seeks to provide additional insight into risk assessment of CLD in aging populations.

## Methods

### Study population

This prospective cohort study utilized data from the baseline survey (2011) and the CLD follow-up data (2015) of CHARLS, a publicly accessible database (http://charls.pku.edu.cn/). The CHARLS, as a nationally representative interdisciplinary longitudinal survey, covered 450 villages or communities across 28 provinces in China, focusing on individuals aged 45 and above. The baseline (wave 1) recruited 17,708 participants, and subsequent surveys were carried out biennially in 2013, 2015, 2018, and 2020 (waves 2–5). The survey collected multi-dimensional data, including basic personal information, family structure, physical well-being evaluations, cognitive assessments, socioeconomic measures, housing conditions, and laboratory examination. This database has become a dependable resource for studying the health conditions of middle-aged and older adults.

**Fig. 1:**
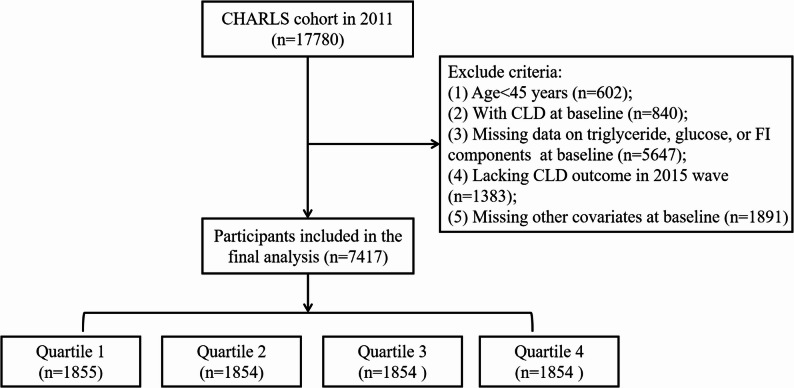
Flowchart of participant selection. Participants were excluded sequentially according to the listed criteria, with the number excluded at each step indicated

Participants were selected from the 2011 and 2015 waves. Exclusion criteria were applied sequentially as follows: (1) age < 45 years at baseline; (2) with a prior diagnosis of CLD at baseline; (3) missing data on key variables required for TyGFI construction, including triglycerides, fasting glucose, or frailty index components (with more than 20% of frailty items missing); (4) lacking CLD outcome data at the 2015 follow-up; (5) missing other covariates at baseline. Ultimately, the final analysis comprised 7,417 participants, categorized into four quartiles (*n* = 1,854-1,855). The participant selection process is shown in Fig. 1.

### Measurement

#### Assessment of TyG-Frailty Index (TyGFI)

The TyG index, calculated from triglyceride and fasting glucose levels, is a widely used surrogate marker of insulin resistance and has been associated with multiple systemic disorders [25]. Its calculation formula was: Ln[triglycerides (mg/dL) × glucose (mg/dL)/2] [7].

The frailty index (FI) was constructed based on the accumulation of 32 health deficits (Supplementary Table 1), encompassing multiple variables, including comorbidities, physical function, disability, depressive symptoms, and cognitive performance [26]. Except for item 32, all items were assigned as binary variables (0 = no deficit; 1 = deficit present). Item 32 was treated as a continuous variable ranging from 0 to 1, with higher values indicating poorer cognitive function. To ensure data quality, participants with more than 20% missing items (> 6 items) were considered to have missing FI data [27].

TyGFI was constructed as the multiplicative product of the TyG index and FI (TyGFI = TyG × FI), with the aim of capturing the combined burden of metabolic dysfunction and physiological vulnerability. This multiplicative formulation has been used in prior epidemiological research to integrate distinct biological domains into a composite exposure index [23, 28]. In this study, the multiplicative formulation was applied to capture the combined burden rather than a formal statistical interaction, as it reflects joint contribution rather than independent additive effects. For comparison, an additive TyG + FI model was also constructed to assess whether the multiplicative formulation provides additional discriminative performance.

#### Assessment of chronic liver disease

CLD was identified based on participants’ self-reported physician diagnoses at each visit, including any liver disease excluding tumors and cancer. Participants were categorized as having CLD (“Yes”) or not having CLD (“No”). This approach has been widely used in CHARLS-based epidemiological studies when clinically adjudicated outcomes are unavailable.

#### Assessment of covariates

Covariates were adjusted for sociodemographic characteristics, health-related behaviors, anthropometric data, and clinical laboratory examination. Covariates were selected based on previous CHARLS-based studies on CLD and research on metabolic dysfunction and frailty [23, 29, 30]. Sociodemographic variables included age, sex, educational attainment (“primary school or below,” “high school,” and “college or above”), location (“city/town” and “village”), and marital status (“married” and “non-married: never married/separated/divorced/widowed”). Health-related behaviors included smoking status (“non-smoker,” “ex-smoker,” and “current smoker”) and drinking status (“more than once a month,” “less than once a month,” and “never”). Data were obtained through trained interviewers utilizing standardized questionnaires. Anthropometric data included systolic blood pressure (SBP), diastolic blood pressure (DBP), and body mass index (BMI). Laboratory indicators consisted of glucose, TG, HbA1c, total cholesterol (TC), high-density lipoprotein cholesterol (HDL-C) and low-density lipoprotein cholesterol (LDL-C).

### Statistical analysis

Continuous variables were shown as mean ± standard deviation (SD), while categorical variables were reported as counts (percentages). Continuous variables were compared using one-way analysis of variance (ANOVA). Categorical variables were compared using the chi-square test. Multivariable logistic regression models were employed to assess the association between TyGFI and incident CLD. TyGFI was analyzed both as a continuous variable (including per IQR increment) and as a categorical variable (quartiles). Four models were constructed: an unadjusted model (Crude Model); Model 1 adjusted for age and sex; Model 2 further adjusted for education, location, marital status, smoking status, drinking status, BMI, SBP, and DBP; and Model 3 additionally adjusted for HbA1c, TC, HDLC, and LDLC. Results were presented as odds ratios (ORs) along with 95% confidence intervals (CIs). Multicollinearity was examined using variance inflation factors (VIFs), with all VIF values below the commonly accepted threshold, indicating no substantial multicollinearity. Nonlinear associations between TyGFI and CLD were assessed using restricted cubic spline (RCS) models with four knots placed at the 5th, 35th, 65th, and 95th percentiles of the TyGFI distribution [31]. Interaction analyses were conducted by incorporating interaction terms [TyGFI × (interaction term)] into the regression models to assess potential effect modification by sociodemographic characteristics, health behaviors, and clinical indicators. Receiver operating characteristic (ROC) curve analysis was performed to evaluate the discriminative performance. The additive TyG + FI model was constructed using identical covariate adjustments. The area under the ROC curve (AUC) values were compared using DeLong tests. All analyses were performed using R software (version 4.5.1).

## Result

### Baseline characteristics

The characteristics of participants are presented in Table 1. A total of 7,417 individuals were included in the baseline analysis (44.63% male; mean age: 58.78 years). Participants were categorized into quartiles (Q1 - Q4) according to TyGFI levels. Overall, individuals in higher TyGFI quartiles tended to be older and exhibited less favorable metabolic profiles. The mean age increased from 55.52 years in Q1 to 62.29 years in Q4. Both SBP and DBP demonstrated an ascending trend across TyGFI quartiles, rising from 124.91/74.39 mmHg in Q1 to 133.44/76.19 mmHg in Q4. Fasting glucose levels increased from 105.44 mg/dL in Q1 to 116.65 mg/dL in Q4, and TG levels rose from 132.13 to 146.86 mg/dL. BMI, HbA1c and TC levels also exhibited modest increases, while HDL-C showed a slight decreasing trend. In addition, smoking, lower educational attainment, and rural residence were more prevalent among individuals with higher TyGFI levels. Notably, drinking frequency decreased across TyGFI quartiles.

**Table 1:** Characteristics of the participants at baseline

	Total(*n* = 7417)	Q1(*n* = 1855)	Q2(*n* = 1854)	Q3(*n* = 1854)	Q4(*n* = 1854)	*p*-value
Sex, *n* (%)						< 0.001
Female	4107(55.37)	866(46.68)	960(51.78)	1069(57.66)	1212(65.37)	
Male	3310(44.63)	989(53.32)	894(48.22)	785(42.34)	642(34.63)	
Age (years)	58.78 ± 9.00	55.52 ± 8.05	58.05 ± 8.58	59.24 ± 8.91	62.29 ± 9.07	< 0.001
Education, n (%)						< 0.001
College or above	184(2.48)	74(3.99)	49(2.64)	39(2.10)	22 (1.19)	
High school	1962(26.45)	727(39.19)	533(28.75)	427(23.03)	275(14.83)	
Primary school or below	5271(71.07)	1054(56.82)	1272(68.61)	1388(74.87)	1557(83.98)	
Location, n (%)						< 0.001
City/town	1171(15.79)	352(18.98)	319(17.21)	262(14.13)	238(12.84)	
Village	6246(84.21)	1503(81.02)	1535(82.79)	1592(85.87)	1616(87.16)	
Marital, n (%)						< 0.001
Married	6544(88.23)	1728(93.15)	1648(88.89)	1641(88.51)	1527(82.36)	
Non-married	873(11.77)	127(6.85)	206(11.11)	213(11.49)	327(17.64)	
Smoking, n (%)						< 0.001
Non-smoker	4648(62.67)	1094(58.98)	1117(60.25)	1177(63.48)	1260(67.96)	
Current smoker	2166(29.20)	647(34.88)	574(30.96)	523(28.21)	422(22.76)	
Ex-smoker	603(8.13)	114(6.15)	163(8.79)	154(8.31)	172(9.28)	
Drinking, n (%)						< 0.001
Drink but less than once a month	604(8.14)	164(9.08)	169(9.12)	143(7.71)	128(6.90)	
Drink more than once a month	1571(21.18)	474(25.55)	430(23.19)	377(20.33)	290(15.64)	
None of these	5242(70.68)	1217(65.61)	1255(67.69)	1334(71.95)	1436(77.45)	
SBP (mmHg)	129.16 ± 21.18	124.91 ± 18.29	128.09 ± 20.23	130.20 ± 22.30	133.44 ± 22.71	< 0.001
DBP (mmHg)	75.20 ± 12.07	74.39 ± 11.36	74.83 ± 12.07	75.38 ± 12.09	76.19 ± 12.68	< 0.001
BMI (kg/m²)	23.58 ± 3.84	23.36 ± 3.39	23.31 ± 3.50	23.74 ± 4.19	23.92 ± 4.18	< 0.001
HbA1c (%)	5.27 ± 0.80	5.16 ± 0.63	5.25 ± 0.79	5.26 ± 0.77	5.40 ± 0.96	< 0.001
Glucose (mg/dL)	110.09 ± 35.81	105.44 ± 25.19	108.36 ± 34.48	109.91 ± 33.35	116.65 ± 46.12	< 0.001
TC (mg/dL)	193.90 ± 38.23	189.95 ± 36.99	194.66 ± 38.88	194.62 ± 38.28	196.38 ± 38.46	< 0.001
TG (mg/dL)	132.13 ± 95.82	120.14 ± 84.29	126.59 ± 93.99	134.91 ± 98.22	146.86 ± 103.72	< 0.001
HDL-C (mg/dL)	51.05 ± 14.94	51.38 ± 14.51	52.18 ± 15.31	51.01 ± 15.02	49.64 ± 14.82	< 0.001
LDL-C (mg/dL)	116.99 ± 35.07	115.48 ± 33.69	117.75 ± 34.99	117.51 ± 35.73	117.21 ± 35.81	0.190
TyG	8.69 ± 0.66	8.57 ± 0.63	8.63 ± 0.66	8.72 ± 0.63	8.84 ± 0.68	< 0.001
Fl	0.13 ± 0.11	0.03 ± 0.02	0.08 ± 0.02	0.14 ± 0.03	0.29 ± 0.10	< 0.001
TyGFI	1.17 ± 0.97	0.25 ± 0.14	0.68 ± 0.13	1.22 ± 0.20	2.53 ± 0.89	< 0.001

Data are presented as mean ± standard deviation or n (%). Non-married: never married, separated, divorced and widowed

*SBP* Systolic blood pressure (mmHg), *DBP* Diastolic blood pressure (mmHg), *BMI* Body mass index (kg/m²), *HbA1c* Glycated hemoglobin (%), *Glucose* fasting glucose (mg/dL), *TC* Total cholesterol (mg/dL), *TG* Fasting triglycerides (mg/dL), *HDLC* Highdensity lipoprotein cholesterol (mg/dL), *LDLC* Lowdensity lipoprotein cholesterol (mg/dL), *TyG* Triglyceride glucose index, *FI* Frailty index: indicator of frailty status, *TyGFI* TyGFrailty index

### Association between TyGFI index and chronic liver disease

The association between TyGFI and CLD was evaluated using multivariable logistic regression models with stepwise adjustment for covariates. As shown in Table 2, a significant and graded association was observed between TyGFI quartiles and CLD risk. In the fully adjusted model (Model 3), compared with Q1, the ORs for CLD were 1.42 (95% CI: 0.94–2.16) in Q2, 1.94 (95% CI: 1.30–2.93) in Q3, and 3.19 (95% CI: 2.16–4.80) in Q4. The *p*-for-trend was statistically significant across all models (*p* < 0.001). When analyzed as a continuous variable, TyGFI remained significantly associated with CLD across all models. Following adjustments for sex, age, educational level, residential area, marital status, lifestyle factors, BMI, blood pressure, glycated hemoglobin, and lipid parameters (Model 3), it was found that each unit increase in TyGFI corresponded to a 38% higher odds of developing new-onset CLD (OR: 1.38, 95% CI: 1.23–1.45). Similarly, each IQR increase in TyGFI was associated with a 4% increase in CLD risk (OR: 1.04, 95% CI: 1.03–1.06).

**Table 2:** Association of TyGFI with the risk of CLD in the CHARLS

	Crude model		Model 1		Model 2		Model 3	
Characteristic	95%CI	*p*	95%CI	*p*	95%CI	*p*	95%CI	*p*
CLD ~ TyGFI	1.31(1.17,1.45)	< 0.001	1.40(1.25,1.56)	< 0.001	1.40(1.25,1.56)	< 0.001	1.38(1.23,1.54)	< 0.001
CLD ~ TyGFI per IQR	1.04(1.02,1.05)	< 0.001	1.05(1.03,1.06)	< 0.001	1.05(1.03,1.06)	< 0.001	1.04(1.03,1.06)	< 0.001
CLD ~ TyGFI(Q1-Q4)								
Q1	ref		ref		ref		ref	
Q2	1.33(0.88,2.01)	0.178	1.44(0.96,2.19)	0.083	1.46(0.96,2.22)	0.077	1.42(0.94,2.16)	0.100
Q3	1.71(1.16,2.55)	0.007	1.95(1.31,2.92)	0.001	1.99(1.34,3.00)	< 0.001	1.94(1.30,2.93)	0.001
Q4	2.55(1.78,3.72)	< 0.001	3.22(2.21,4.78)	< 0.001	3.30(2.24,4.94)	< 0.001	3.19(2.16,4.80)	< 0.001
*p* for trend		< 0.001		< 0.001		< 0.001		< 0.001

Crude model: Unadjusted

Model 1: Adjusted for age and sex

Model 2: Further adjusted for educational level, location, marital status, smoking status, drinking status, BMI, SBP, and DBP

Model 3: Fully adjusted, additionally including HbA1c, TC, HDL-C, and LDL-C

**Fig. 2:**
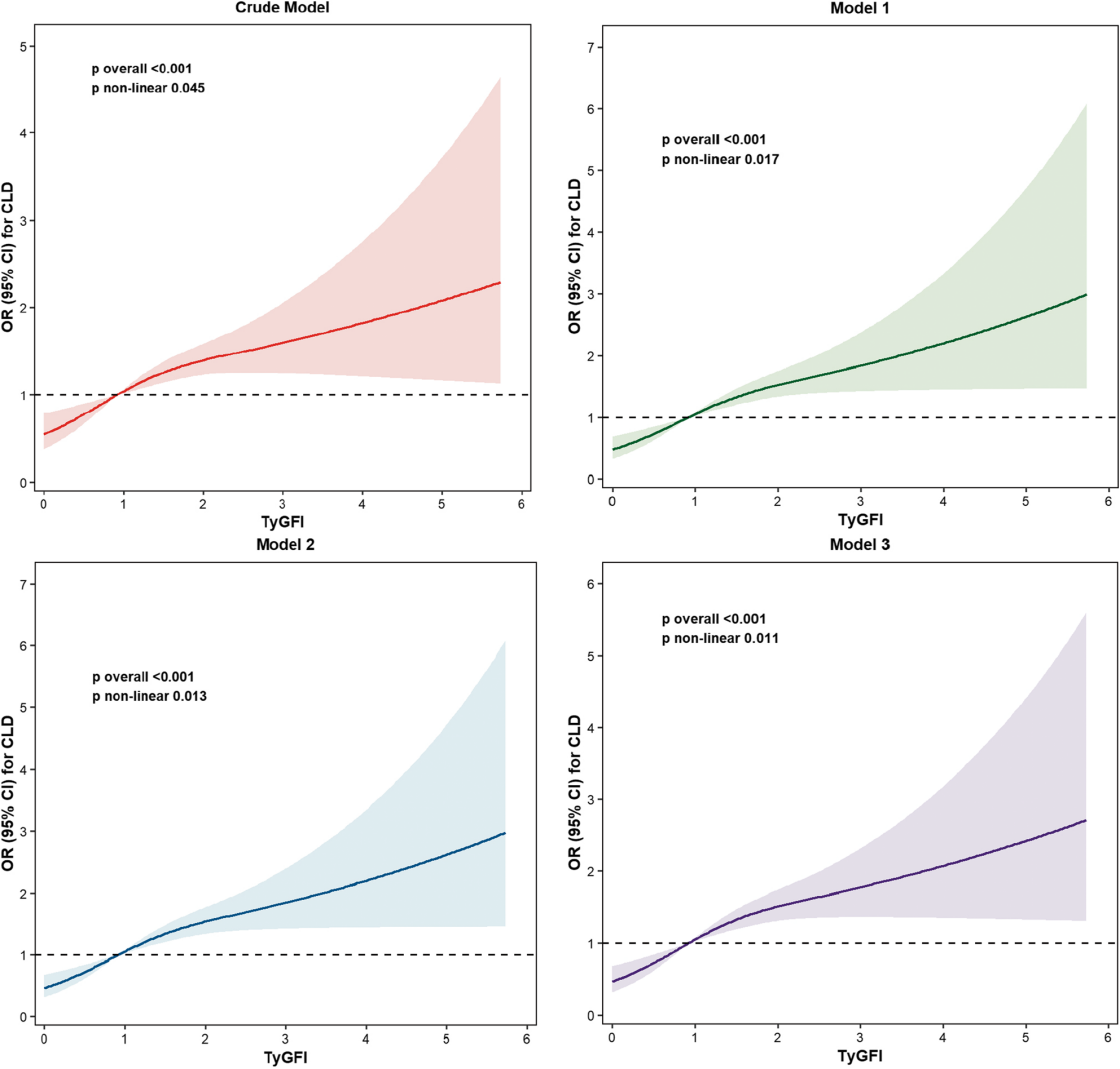
A dose–response curve based on RCS analysis demonstrates the relationship between TyGFI and CLD risk.The shaded area represents the 95% confidence interval

The RCS analyses (Fig. 2) demonstrated a nonlinear positive correlation between the two variables, with consistent trends across all adjustment models. The RCS analysis results further supporting the findings of the quartile-based analysis.

### ROC analysis

**Fig. 3:**
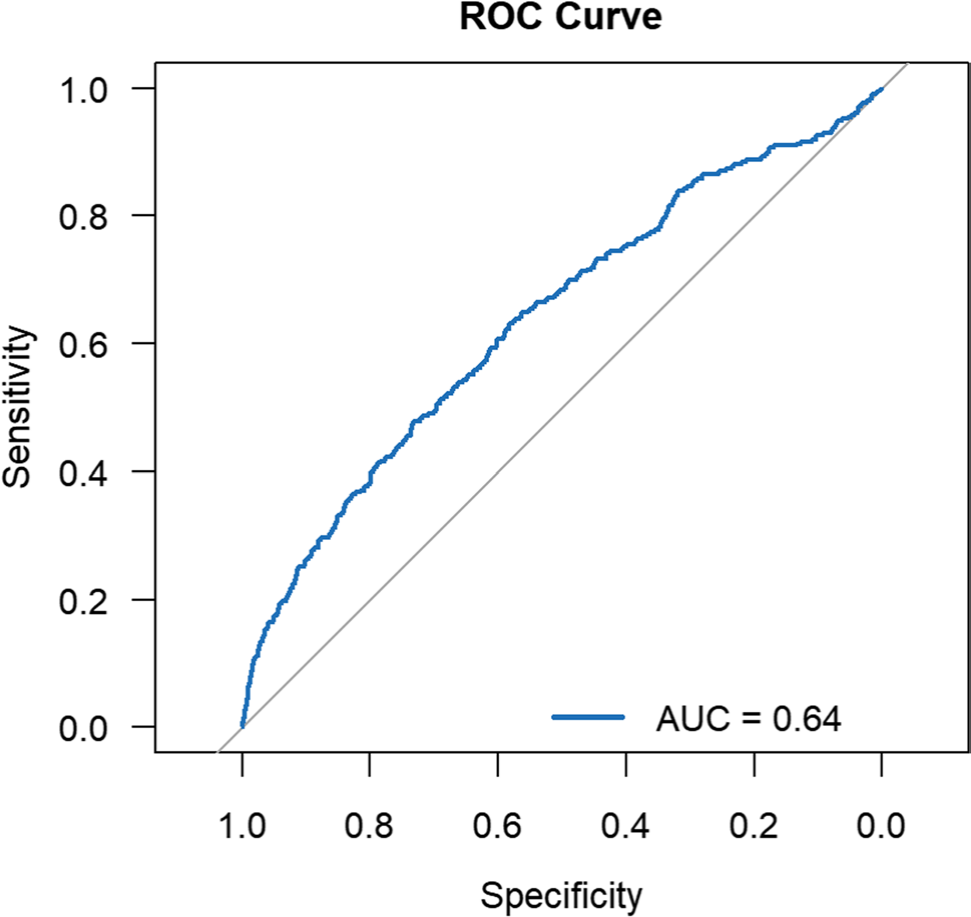
Receiver operating characteristic (ROC) curve of TyGFI for CLD

To evaluate the discriminative performance of TyGFI for CLD, ROC curve analysis was conducted. As shown in Fig. 3, TyGFI demonstrated moderate discriminative ability, with an AUC of 0.64 (95% CI: 0.61–0.67). In comparative analyses, the AUC of TyGFI was higher than that of the TyG index (AUC: 0.55, 95% CI: 0.51–0.59) and the FI (AUC: 0.59, 95% CI: 0.55–0.62). An additive model (TyG + FI) constructed under identical covariate adjustments yielded an AUC of 0.57 (95% CI: 0.53–0.60). DeLong tests showed that the differences in AUC between TyGFI and TyG (*p* < 0.001) ,TyGFI and FI (*p* < 0.05), and TyGFI and the additive model (*p* < 0.05) were statistically significant.ROC curves for TyG, FI, and the additive model (TyG + FI) are presented in Supplementary Figs. 1–3.

### Subgroup analysis

**Fig. 4:**
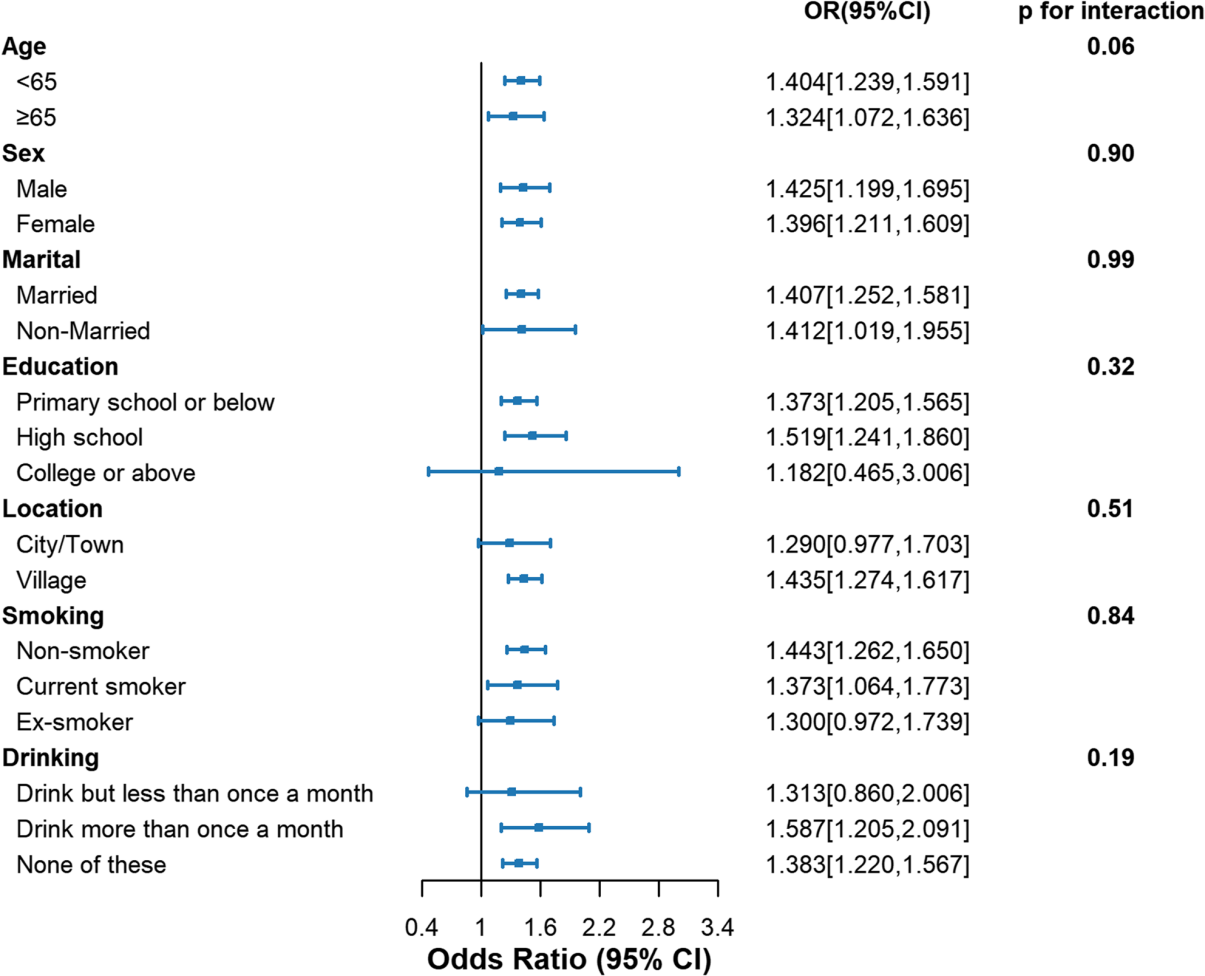
Forest plot of stratified analysis of the association of TyGFI with the risk of CLD. OR, odds ratio; CI, confidence interval. Non-married: never married, separated, divorced and widowed

To further investigate the relationship between TyGFI and the risk of CLD, subgroup and interaction analyses were conducted based on age, sex, marital status, education level, location, smoking status, and alcohol consumption (Fig. 4). No significant interaction effects were detected (all *p* for interaction > 0.05), indicating no evidence of heterogeneity in the association between TyGFI and CLD across the examined subgroups.

### Sensitivity analyses

Several sensitivity analyses were conducted to assess the robustness of the findings. Baseline characteristics of included versus excluded participants were compared to evaluate potential selection bias (Supplementary Table 2). After excluding extreme outliers, TyGFI remained significantly associated with CLD (OR: 1.55, 95% CI: 1.34–1.78) (Supplementary Table 3). To address potential outcome misclassification, cases were redefined to include both self-reported CLD and individuals receiving liver disease–related medications. The results remained consistent (OR: 1.38, 95% CI: 1.23–1.54) (Supplementary Table 4). Overall, these analyses support the robustness of the observed relationship between TyGFI and CLD.

## Discussion

Based on the CHARLS database, we first systematically investigated the association between TyGFI and the risk of CLD in this cohort study. Our results demonstrate a significant association between elevated TyGFI levels and an increased risk of CLD. After adjusting for confounders, each unit increase in TyGFI was associated with a 38% higher odds of incident CLD during follow-up. Participants in the highest quartile of TyGFI exhibited a markedly greater risk of CLD. RCS analysis further revealed a nonlinear dose-response relationship. In ROC analyses, TyGFI showed statistically significant differences in AUC compared with TyG index, FI, and the additive model (TyG + FI). With an AUC of 0.64, its discrimination ability remains moderate and does not support its use as a standalone screening tool, but it may offer complementary information when used alongside established clinical markers. Subgroup analyses revealed no significant interaction effects, suggesting that the association between TyGFI and CLD did not differ statistically across different subgroups. Sensitivity analyses, including outlier exclusion and restriction to medication-confirmed cases, produced consistent results, supporting the robustness of the findings. In addition, analyses treating TyGFI as both a continuous and categorical variable yielded consistent results. Taken together, these findings suggest that TyGFI is associated with CLD risk and may reflect the combined metabolic and functional burden relevant to disease development.

Our findings are consistent with previous studies while providing additional insights. Numerous studies have established the TyG index as a simple and reliable surrogate marker of insulin resistance [32]. Increasing evidence indicates that insulin resistance and impaired glucose tolerance are common in NAFLD/MAFLD, cirrhosis, and other chronic liver diseases [33]. Nayak et al. reported that patients with NAFLD exhibit a significantly elevated TyG index (OR = 2.36, 95% CI: 1.88–2.97) [34]. Multiple studies have confirmed that TyG index is a reliable and non-invasive indicator for assessing NAFLD risk; monitoring its dynamic change may facilitate the early detection and intervention [35–38]. A meta-analysis indicated that TyG index is effective in accurately diagnosing and predicting MAFLD (AUC = 0.75) [39]. Additionally, the association between TyG and hepatic fibrosis has also been well documented. Elevated TyG levels have been associated with both increased fibrosis risk and fibrosis progression in NAFLD patients, suggesting a potential link between metabolic dysfunction and CLD progression [40, 41]. Moreover, patients with chronic viral hepatitis often experience long-term metabolic disturbances. Mechanistic research has shown that the hepatitis B virus X protein (HBx) interferes with insulin signaling by reducing insulin receptor substrate-1 (IRS1), thereby promoting insulin resistance [42]. Consistently, Yang et al. identified TyG index as an independent risk factor for hepatocellular carcinoma in patients with HBV-related cirrhosis (OR = 2.62, 95% CI: 1.32–5.20) [43]. Although prior studies have primarily focused on the association between the TyG index and specific liver disease subtypes, such as NAFLD/MAFLD, chronic hepatitis B, or liver fibrosis, its predictive performance across the entire spectrum of chronic liver diseases remains underexplored. Our study extends previous work by examining the association and discriminative performance of TyGFI across a broader spectrum of CLD.

Multiple prior investigations have also demonstrated the association between frailty and CLD. Zhong et al. reported that frailty was associated with an increased likelihood of developing CLD, suggesting that frailty assessment may help identify high-risk populations [17]. Laube et al. further showed that frailty predicted adverse prognosis in advanced liver disease and was associated with heightened morbidity and mortality [44]. Consistent with these findings, recent evidence indicates that functional dependence, as a related manifestation of physiological vulnerability, is associated with an increased risk of adverse health outcomes among middle-aged and older adults [45]. The FI, as a comprehensive indicator of physiological deterioration, is therefore relevant for understanding not only liver-specific outcomes but also broader health risks. However, most previous studies have focused on the independent effects of either TyG index or FI, with limited exploration of their combined influence. Building upon this evidence, our study expands prior findings by showing that the combined presence of metabolic dysfunction and frailty is associated with an increased risk of CLD.

Although direct mechanistic evidence was not available in the present study, a hypothetical interpretation based on existing literature. Previous studies have suggested that insulin resistance may contribute to hepatocellular injury and fibrosis through pathways involving inflammation, oxidative stress, and mitochondrial dysfunction [46]. Impaired insulin signaling specifically reduces the activity of crucial mitochondrial regulators, such as SIRT1 and PGC-1α, leading to disrupted energy homeostasis in hepatocytes [47, 48]. Meanwhile, the decline in physiological reserve captured by the frailty index has been linked to mitochondrial dysfunction and enhanced inflammatory responses, potentially increasing vulnerability to metabolic stressors [15]. In this context, the coexistence of metabolic dysfunction and frailty may be associated with processes such as hepatic stellate cell activation and pro-fibrotic pathways, thereby contributing to hepatocellular injury [47, 49]. Taken together, the combined burden reflected by the TyG index and FI may help explain the observed association between TyGFI and CLD through pathways related to insulin resistance, inflammation, and oxidative stress. However, due to the lack of virological markers and liver enzyme indicators in the CHARLS data, the specific etiologies of CLD could not be distinguished; therefore, these mechanistic considerations should be interpreted as general, non–etiology-specific biological explanations rather than causal inferences.

This study introduces the TyGFI, a composite risk indicator that integrates the surrogate marker of insulin resistance, the TyG index, with the FI, an effective measure of cumulative physiological deficits. TyGFI reflects the combined burden of these interrelated processes rather than a direct measure of biological interaction. Although both TyG and FI have been independently associated with increased CLD risk [17, 35–38], their integration provides an additional perspective for evaluating risk in aging populations. Moreover, this approach also addresses the limitations of previous studies that focused solely on metabolic markers (such as TyG-WHtR and TyG-BMI) [13, 42, 50], while overlooking the functional health status. By incorporating both metabolic and functional dimensions, TyGFI may offer a more comprehensive framework for risk assessment of CLD. Methodologically, this study benefits from the use of a large, nationally representative prospective cohort (CHARLS), enhancing the generalizability of the findings. We constructed three statistical models adjusting for multiple covariates, such as demographic, lifestyle, and clinical factors, to minimize potential confounding bias. In addition, multiple analytical strategies—including multivariable logistic regression, RCS, ROC, subgroup, and sensitivity analyses—were applied to ensure the robustness of the results. Overall, this study highlights the potential relevance of integrating metabolic and functional measures in CLD research. The multidimensional nature of TyGFI may provide complementary information for risk stratification in aging populations.

From a practical perspective, the cost-benefit ratio of incorporating frailty assessment into routine risk evaluation should be carefully considered. Although TyGFI demonstrated statistically significant improvement in discrimination, the absolute AUC gain was modest. Given that the frailty index was derived from a multi-item assessment, its routine implementation in clinical practice may be challenging. Future studies should explore simplified versions of the TyG–Frailty index using fewer functional items to enhance feasibility.

Several limitations of this study should be acknowledged. First, although the CHARLS dataset provides prospective follow-up data, the exact timing of CLD onset was unavailable, precluding time-to-event analyses such as Cox proportional hazards regression. Accordingly, multivariable logistic regression was used to estimate cumulative risk rather than hazard-based associations. Therefore, the findings should be interpreted as cumulative risk estimates over the follow-up period rather than time-dependent hazard ratios. Future studies with precise outcome timing are warranted to enable survival analyses. Second, both the frailty index and CLD outcomes were based on self-reported data, which may introduce measurement error and misclassification. Such misclassification is unlikely to be related to TyGFI levels and would most likely bias the observed associations toward the null. In addition, sensitivity analyses restricted to medication-confirmed cases produced comparable effect estimates, suggesting that potential misclassification did not materially alter the results. Future studies incorporating imaging or laboratory biomarkers are needed to improve outcome ascertainment. Third, as an observational study, causal inference is inherently limited despite adjustment for multiple covariates. Residual confounding from unmeasured factors, such as genetic susceptibility, environmental exposures, and dietary patterns, cannot be excluded. Future research could strengthen causal inference through approaches such as Mendelian randomization. Fourth, although the CHARLS cohort is nationally representative of middle-aged and older adults in China, the generalizability of these findings to populations with different genetic backgrounds and healthcare systems necessitates further investigation. Finally, although the multiplicative formulation appears to capture joint effects more effectively than a simple additive combination, its incremental benefit is modest and requires further validation in independent cohorts.

## Conclusion

TyGFI was independently associated with incident CLD and demonstrated modest discriminative ability. As a composite index integrating metabolic and functional dimensions, it may help identify individuals at increased risk of CLD, particularly in aging populations. Although not intended as a standalone screening tool, TyGFI may potentially complement existing non-invasive approaches when used alongside established clinical markers. Further validation in independent populations is warranted.

## Supplementary Information

Supplementary Material 1.

Supplementary Material 2.

Supplementary Material 3.

Supplementary Material 4.

Supplementary Material 5.

## Data Availability

The data analyzed during the current study are available in the official website of the China Health and Retirement Longitudinal Study (CHRLS) (http://charls.pku.edu.cn). The original data during the current study are available from the corresponding author on reasonable request.
