# Supplementary material for: The association of the combined triglyceride-glucose and frailty index with chronic liver disease: evidence from the CHARLS study
Source: BMC Gastroenterol. 2026 Apr 15;26:317. doi: 10.1186/s12876-026-04818-1 (PMC13188412; doi:10.1186/s12876-026-04818-1)
Supplement: Supplementary file 1 — Supplementary Material 1. [file 12876_2026_4818_MOESM1_ESM.docx]

**Supplementary Table 1. Detailed description of the 32 items used to construct the frailty index in CHARLS and their corresponding cut-off values.**

| No | Description of the items | Cut-off value |
| --- | --- | --- |
| 1 | Self-reported physician diagnosed hypertension | Yes = 1, No = 0 |
| 2 | Self-reported physician diagnosed diabetes | Yes = 1, No = 0 |
| 3 | Self-reported diagnosis of heart attack, coronary heart disease, angina, congestive heart failure, or other heart problems | Yes = 1, No = 0 |
| 4 | Self-reported diagnosis of stroke | Yes = 1, No = 0 |
| 5 | Self-reported physician diagnosed cancer | Yes = 1, No = 0 |
| 6 | Self-reported physician diagnosed arthritis | Yes = 1, No = 0 |
| 7 | Self-reported physician diagnosed chronic lung disease | Yes = 1, No = 0 |
| 8 | Self-reported physician diagnosed asthma | Yes = 1, No = 0 |
| 9 | Self-reported physician diagnosed any emotional, nervous, or psychiatric problems | Yes = 1, No = 0 |
| 10 | Self-reported physician diagnosed memory-related disease | Yes = 1, No = 0 |
| 11 | Self-reported vision problems | Yes = 1, No = 0 |
| 12 | Self-reported hearing problems | Yes = 1, No = 0 |
| 13 | Self-reported general health status | Poor or fair = 1, excellent, very good, or good = 0 |
| 14 | Difficulty with dressing | Yes = 1, No = 0 |
| 15 | Difficulty with bathing or showering | Yes = 1, No = 0 |
| 16 | Difficulty with eating | Yes = 1, No = 0 |
| 17 | Difficulty with getting in and out of bed | Yes = 1, No = 0 |
| 18 | Difficulty with using the toilet | Yes = 1, No = 0 |
| 19 | Difficulty with managing money | Yes = 1, No = 0 |
| 20 | Difficulty with taking medications | Yes = 1, No = 0 |
| 21 | Difficulty with shopping for groceries | Yes = 1, No = 0 |
| 22 | Difficulty with preparing meals | Yes = 1, No = 0 |
| 23 | Difficulty with doing housework | Yes = 1, No = 0 |
| 24 | Mobility: difficulty with walking 100 yards | Yes = 1, No = 0 |
| 25 | Mobility: difficulty with getting up from a chair after sitting for long periods | Yes = 1, No = 0 |
| 26 | Mobility: difficulty with climbing several flights of stairs without resting | Yes = 1, No = 0 |
| 27 | Mobility: difficulty with lifting or carrying weights over 10 pounds/jins | Yes = 1, No = 0 |
| 28 | Mobility: difficulty with picking up a coin from the table | Yes = 1, No = 0 |
| 29 | Mobility: difficulty with stooping, kneeling, or crouching | Yes = 1, No = 0 |
| 30 | Mobility: difficulty with reaching arms above shoulder level | Yes = 1, No = 0 |
| 31 | Depression: CESD-10 questionnaire | CESD-10 >10 =1, ≤10 =0 in the CHARLS |
| 32 | Cognition: (memory test score + orientation test score) **/** 14 | Continuous, ranging from 0 to 1 |

Memory-related disease indicates Alzheimer’s disease or dementia, organic brain senility, or other serious memory impairment.

Depression is evaluated using Center for Epidemiologic Studies Depression Scale (CESD). In the CHARLS, CESD-10 is used, and the total score ranges from 0 to 30. The higher score indicates more severe depressive symptoms.

The memory score is the average of words that are not recalled in the immediate and delayed word recall tasks. The memory score ranges from 0 to 10. The orientation test comprises four questions about the day of the week, the month, the date of the month, and the year. One point is given for each wrong answer, and the range is from 0 to 4.
