# Supplementary material for: The association of the combined triglyceride-glucose and frailty index with chronic liver disease: evidence from the CHARLS study
Source: BMC Gastroenterol. 2026 Apr 15;26:317. doi: 10.1186/s12876-026-04818-1 (PMC13188412; doi:10.1186/s12876-026-04818-1)
Supplement: Supplementary file 2 — Supplementary Material 2. [file 12876_2026_4818_MOESM2_ESM.docx]

**Supplementary Table 2. Sensitivity Analysis: Baseline Differences Between Included and Excluded Participants**

|  | **Total(n=13613)** | **Included(n=7417)** | **Excluded(n=6196)** | ***p*-vaule** |
| --- | --- | --- | --- | --- |
| **Sex, n (%)** |  |  |  | <0.001 |
| Female | 7251 (53.27) | 4107(55.37) | 3144 (50.74) |  |
| Male | 6362(46.73) | 3310(44.63) | 3052 (49.26) |  |
| **Age (years)** | 58.63 ± 9.94 | 58.78 ± 9.00 | 58.45 ± 10.97 | 0.057 |
| **Education, n (%)** |  |  |  | <0.001 |
| College or above | 488(3.59) | 184(2.48) | 304(4.91) |  |
| High school | 3734 (27.44) | 1962 (26.45) | 1772 (28.61) |  |
| Primary school or below | 9388(68.98) | 5271 (71.07) | 4117 (66.48) |  |
| **Location, n (%)** |  |  |  | <0.001 |
| City/town | 2604 (19.14) | 1171 (15.79) | 1433 (23.16) |  |
| Village | 11000 (80.86) | 6246(84.21) | 4754 (76.84) |  |
| **Marital , n (%)** |  |  |  | <0.001 |
| Married | 11831 (86.92) | 6544(88.23) | 5287(85.34) |  |
| Non-married | 1781 (13.08) | 873(11.77) | 908 (14.66) |  |
| **Smoking, n (%)** |  |  |  | <0.001 |
| Non-smoker | 8246(60.96) | 4648 (62.67) | 3598 (58.88) |  |
| Current smoker | 4100 (30.31) | 603 (8.13) | 579(9.47) |  |
| Ex-smoker | 1182 (8.74) | 603(8.12) | 788(9.02) |  |
| **Drinking, n (%)** |  |  |  | 0.054 |
| Drink but less than once a  month | 1070(8.29) | 604(8.14) | 466(8.49) |  |
| Drink more than once a month | 2640 (20.45) | 1571 (21.18) | 1069 (19.46) |  |
| None of these | 9199 (71.26) | 5242 (70.68) | 3957(72.05) |  |
| **SBP (mmHg)** | 129.46 ± 21.48 | 129.16 ± 21.18 | 129.82 ± 21.84 | 0.074 |
| **DBP (mmHg)** | 75.46 ± 12.25 | 75.20 ± 12.07 | 75.78 ± 12.45) | 0.006 |
| **BMI (kg/m²)** | 23.46 ± 3.89 | 23.58 ± 3.84 | 23.32 ± 3.93 | <0.001 |
| **HbA1c (%)** | 5.26 ± 0.82 | 5.27 ± 0.80 | 5.26 ± 0.87 | 0.527 |
| **Glucose (mg/dL)** | 110.40 ± 37.56 | 110.09 ± 35.81 | 111.39 ± 42.62 | 0.146 |
| **TC (mg/dL)** | 193.50 ± 39.01 | 193.90 ± 38.23 | 192.23 ± 41.35 | 0.070 |
| **TG (mg/dL)** | 133.67 ± 109.88 | 132.13 ± 95.82 | 138.53 ± 145.37 | 0.014 |
| **HDL-C (mg/dL)** | 51.08 ± 15.30 | 51.05 ± 14.94 | 51.17 ± 16.36 | 0.738 |
| **LDL-C (mg/dL)** | 116.37 ± 35.12 | 116.99 ± 35.07 | 114.44 ± 35.23 | 0.002 |
| **TyG** | 8.69 ± 0.68 | 8.69 ± 0.66 | 8.69 ± 0.73 | 0.801 |
| **Fl** | 0.14 ± 0.12 | 0.13 ± 0.11 | 0.14 ± 0.12 | 0.079 |

**Note:**The exclusion criteria for this study were as follows: (1) Participants with a baseline age below 45 years; (2) with a history of chronic liver disease at baseline; (3) missing measurements for triglycerides, glucose, or FI components, as well as any other covariates; (4) with missing data for outcome variables related to chronic liver disease. Ultimately, a total of 7,417 participants were included in the analysis, while 6,196 individuals were excluded.
