# Supplementary material for: The association of the combined triglyceride-glucose and frailty index with chronic liver disease: evidence from the CHARLS study
Source: BMC Gastroenterol. 2026 Apr 15;26:317. doi: 10.1186/s12876-026-04818-1 (PMC13188412; doi:10.1186/s12876-026-04818-1)
Supplement: Supplementary file 3 — Supplementary Material 3. [file 12876_2026_4818_MOESM3_ESM.docx]

**Supplementary Table 3. Sensitivity Analysis: Associations Between TyGFI and Risk of CLD After Outlier Exclusion**

| **Character** | **Crude model** | | **Model 1** | | **Model 2** | | **Model 3** | |
| --- | --- | --- | --- | --- | --- | --- | --- | --- |
|  | **95%CI** | ***p*** | **95%CI** | ***p*** | **95%CI** | ***p*** | **95%CI** | ***p*** |
| CLD ~ TyGFI | 1.41(1.24,1.60) | <0.001 | 1.55(1.35,1.78) | <0.001 | 1.56(1.35,1.79) | <0.001 | 1.55(1.34,1.78) | <0.001 |
| CLD ~ TyGFI per IQR | 1.05(1.03,1.07) | <0.001 | 1.06(1.04,1.08) | <0.001 | 1.05(1.04,1.08) | <0.001 | 1.06(1.04,1.08) | <0.001 |
| CLD ~ TyGFI(Q1-Q4) | | | | | | | | |
| Q1 | ref |  | ref |  | ref |  | ref |  |
| Q2 | 1.33(0.88,2.01) | 0.178 | 1.45(0.96,2.20) | 0.080 | 1.46(0.96,2.22) | 0.076 | 1.44(0.95,2.19) | 0.088 |
| Q3 | 1.71(1.16,2.55) | 0.007 | 1.96(1.32,2.94) | <0.001 | 2.00(1.34,3.02) | <0.001 | 1.98(1.33,2.98) | <0.001 |
| Q4 | 2.56(1.78,3.75) | <0.001 | 3.26(2.22,4.86) | <0.001 | 3.31(2.24,4.98) | <0.001 | 3.27(2.20,4.93) | <0.001 |
| *p* for trend |  | <0.001 |  | <0.001 |  | <0.001 |  | <0.001 |

**Note:** To mitigate the impact of extreme values, we employed a two-step outlier exclusion strategy: (1) applying first and 99th percentile truncation to all continuous covariates to reduce the impact of distribution tails; (2) excluding participants whose TyGFI values exceeded the mean ± 3 standard deviations.

Crude model: Unadjusted.

Model 1: Adjusted for age and sex.

Model 2: Further adjusted for educational level, location, marital status, smoking status, drinking status, BMI, SBP, and DBP.

Model 3: Fully adjusted, additionally including HbA1c, TC, HDL-C, and LDL-C.
