# Supplementary material for: The association of the combined triglyceride-glucose and frailty index with chronic liver disease: evidence from the CHARLS study
Source: BMC Gastroenterol. 2026 Apr 15;26:317. doi: 10.1186/s12876-026-04818-1 (PMC13188412; doi:10.1186/s12876-026-04818-1)
Supplement: Supplementary file 4 — Supplementary Material 4. [file 12876_2026_4818_MOESM4_ESM.docx]

**Supplementary Table 4. Sensitivity Analysis: Associations Between TyGFI and Risk of CLD (based on relevant medication use and self-reported cases)**

| **Character** | **Crude model** | | **Model 1** | | **Model 2** | | **Model 3** | |
| --- | --- | --- | --- | --- | --- | --- | --- | --- |
|  | **95%CI** | ***p*** | **95%CI** | ***p*** | **95%CI** | ***p*** | **95%CI** | ***p*** |
| CLD ~ TyGFI | 1.31(1.18,1.45) | <0.001 | 1.40(1.26,1.56) | <0.001 | 1.40(1.25,1.56) | <0.001 | 1.38(1.23,1.54) | <0.001 |
| CLD ~ TyGFI per IQR | 1.04(1.02,1.05) | <0.001 | 1.05(1.03,1.06) | <0.001 | 1.05(1.03,1.06) | <0.001 | 1.04(1.03,1.06) | <0.001 |
| CLD ~ TyGFI(Q1-Q4) | | | | | | | | |
| Q1 | ref |  | ref |  | ref |  | ref |  |
| Q2 | 1.32(0.88,1.99) | 0.181 | 1.44(0.96,2.17) | 0.082 | 1.45(0.96,2.19) | 0.079 | 1.41(0.94,2.14) | 0.102 |
| Q3 | 1.67(1.14,2.48) | 0.009 | 1.91(1.29,2.85) | 0.001 | 1.95(1.31,2.92) | 0.001 | 1.90(1.28,2.85) | 0.002 |
| Q4 | 2.54(1.78,3.69) | <0.001 | 3.24(2.23,4.97) | <0.001 | 3.30(2.25,4.92) | <0.001 | 3.20(2.17,4.78) | <0.001 |
| *p* for trend |  | <0.001 |  | <0.001 |  | <0.001 |  | <0.001 |

**Note:** CLD is defined as participants who self-report having CLD and those taking medications related to liver disease.

Crude model: Unadjusted.

Model 1: Adjusted for age and sex.

Model 2: Further adjusted for educational level, location, marital status, smoking status, drinking status, BMI, SBP, and DBP.

Model 3: Fully adjusted, additionally including HbA1c, TC, HDL-C, and LDL-C.
