# Supplementary material for: The association of the combined triglyceride-glucose and frailty index with chronic liver disease: evidence from the CHARLS study
Source: BMC Gastroenterol. 2026 Apr 15;26:317. doi: 10.1186/s12876-026-04818-1 (PMC13188412; doi:10.1186/s12876-026-04818-1)
Supplement: Supplementary file 5 — Supplementary Material 5. [file 12876_2026_4818_MOESM5_ESM.docx]

**Supplementary Figure**

**
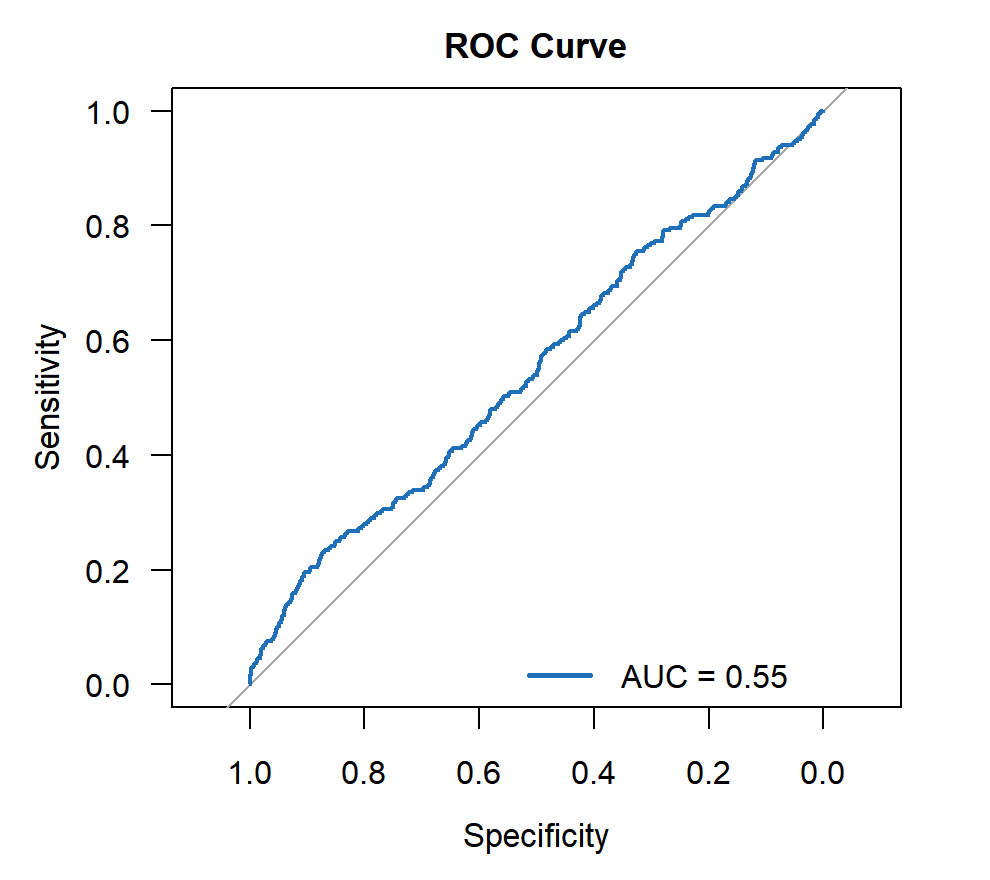
**

**Supplementary Figure 1.** Receiver operating characteristic (ROC) curve of the TyG index for CLD.


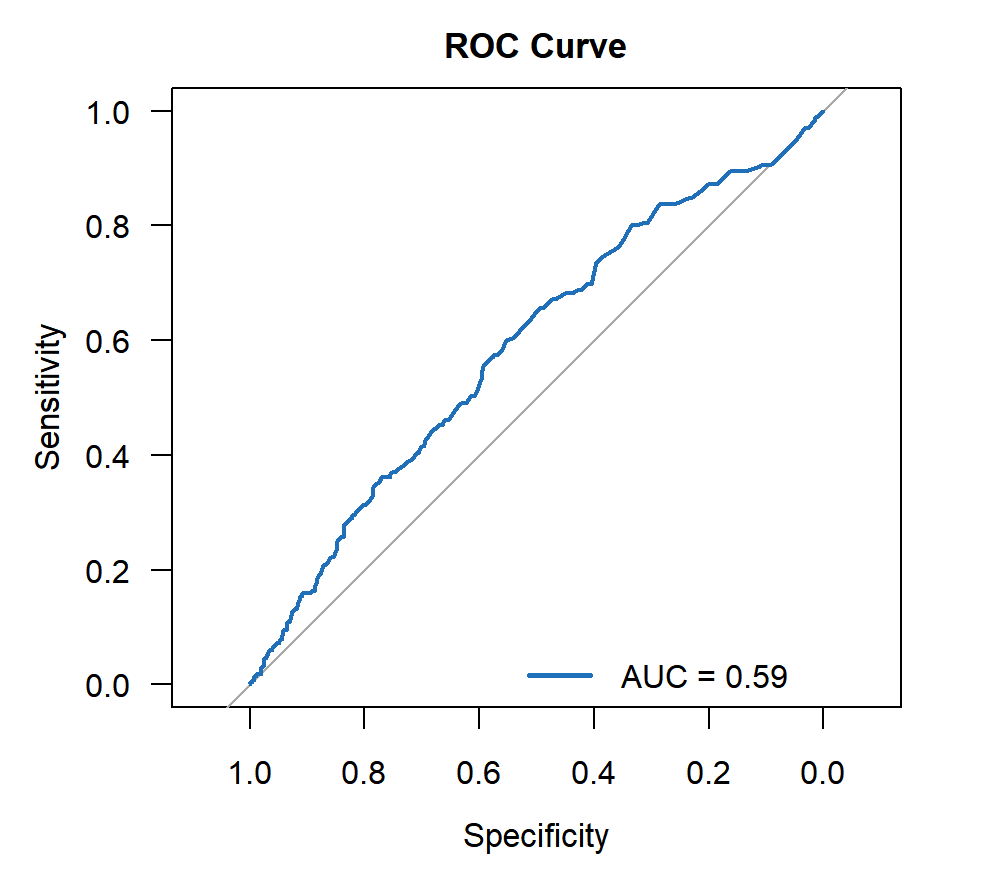


**Supplementary Figure 2.** Receiver operating characteristic (ROC) curve of the frailty index for CLD.


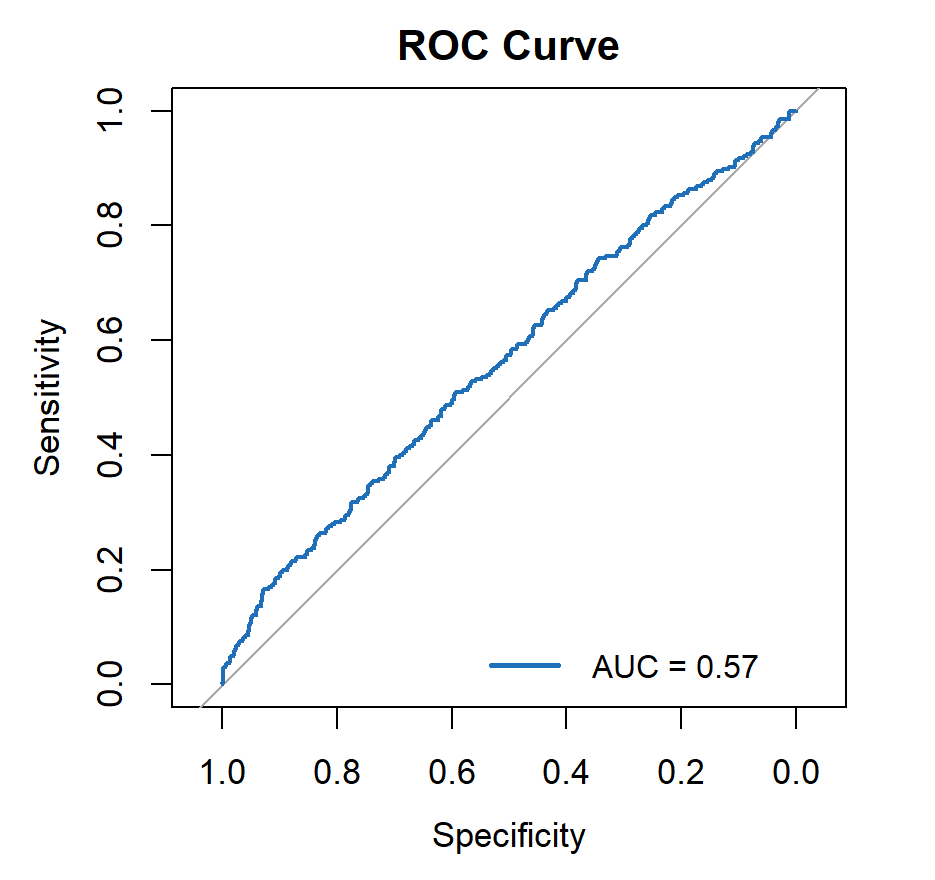


**Supplementary Figure 3.** Receiver operating characteristic (ROC) curve of the additive model (TyG + FI) for CLD.
